# Supplementary material for: Quantification of flexoelectricity in PbTiO3/SrTiO3 superlattice polar vortices using machine learning and phase-field modeling
Source: Nat Commun. 2017 Nov 13;8:1468. doi: 10.1038/s41467-017-01733-8 (PMC5684141; doi:10.1038/s41467-017-01733-8)
Supplement: Supplementary file 1 — Supplementary Information [file 41467_2017_1733_MOESM1_ESM.pdf]

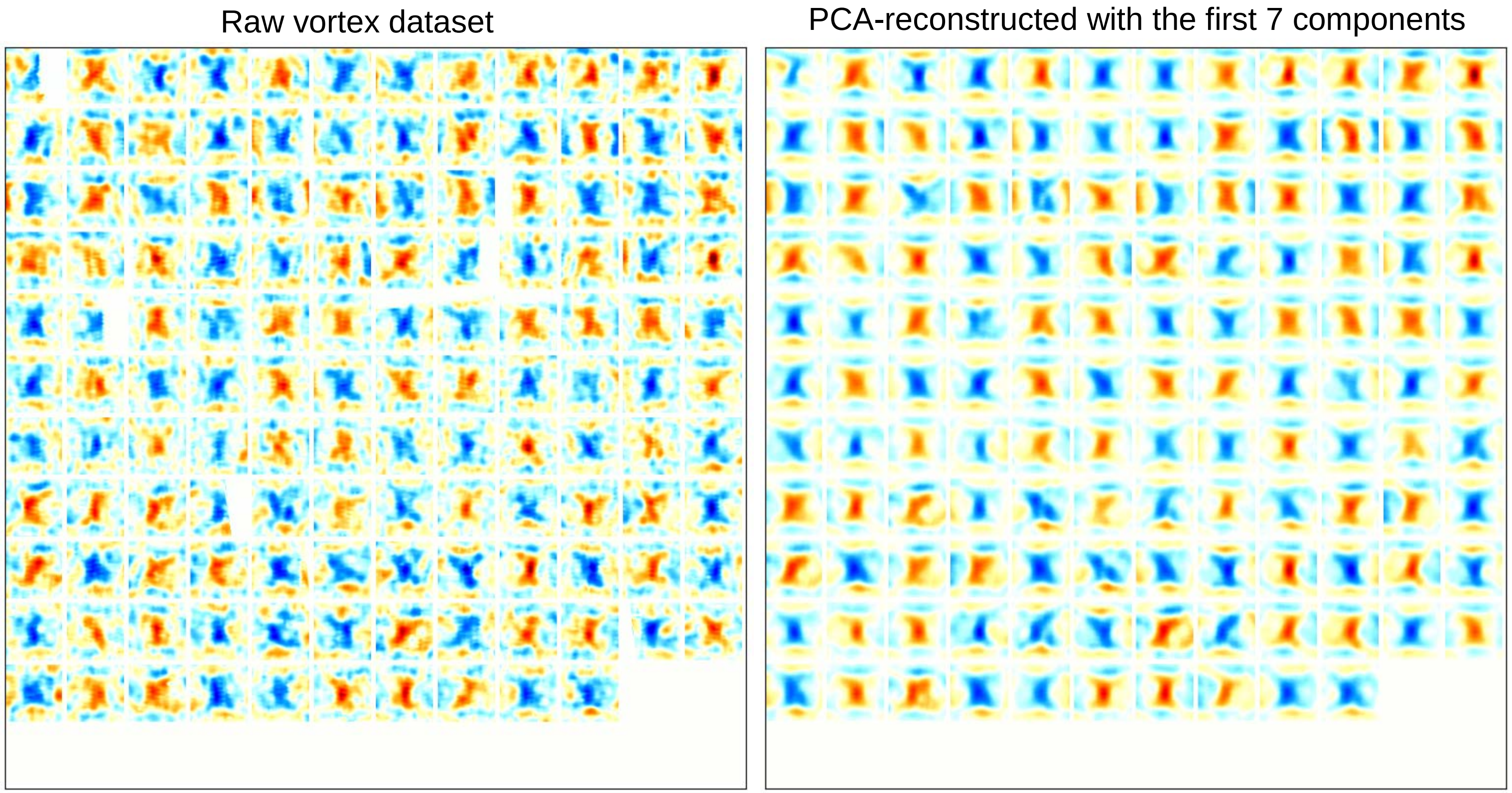

Supplementary Figure 1 | The entire vortex dataset of $(PbTiO_3)_{10}/(SrTiO_3)_{10}$ superlattices.

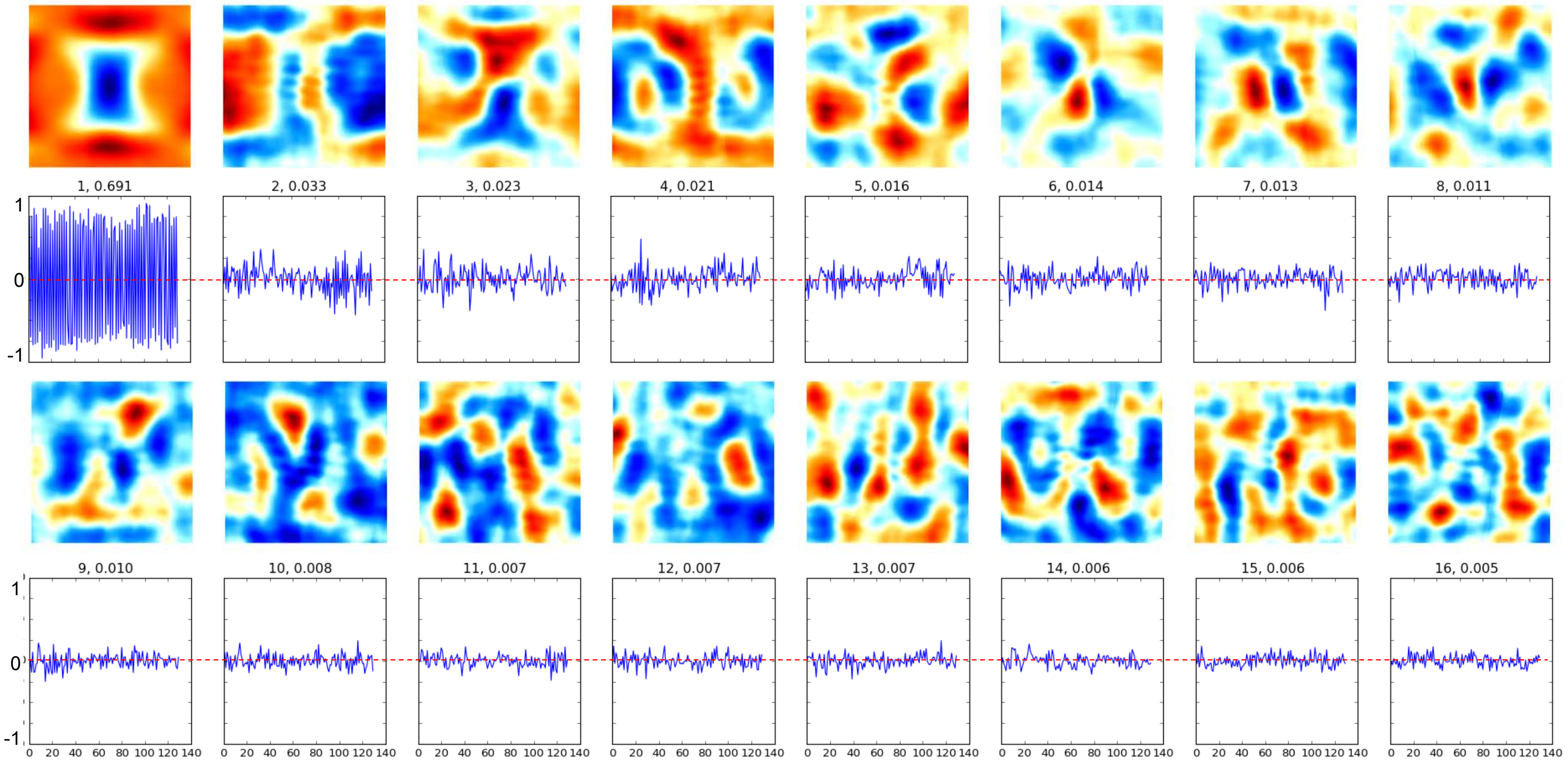

Supplementary Figure 2 | The first 16 PCA components of the vortex dataset: 1st and 3rd rows, loading maps; 2nd and 4th rows, eigenvectors. The variance ratio is shown after each component number.

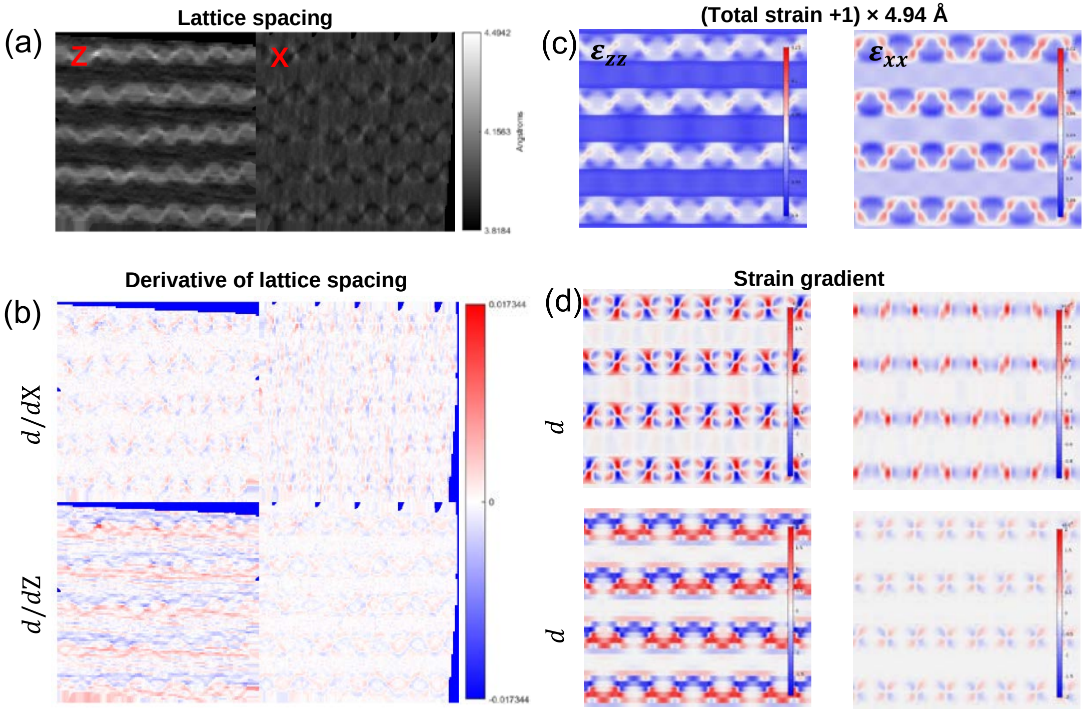

Supplementary Figure 3 | Strain (**a**, **c**) and strain gradient (**b**, **d**) distributions in the $(PTO)_{10}/(STO)_{10}$ superlattices, yielded from the STEM measurement (**a**, **b**) and phase-field modeling (**c**, **d**). The model corresponds to the best-fit flexocoupling conditions. Note that the strain is represented as lattice spacings relative to that of DSO (001).

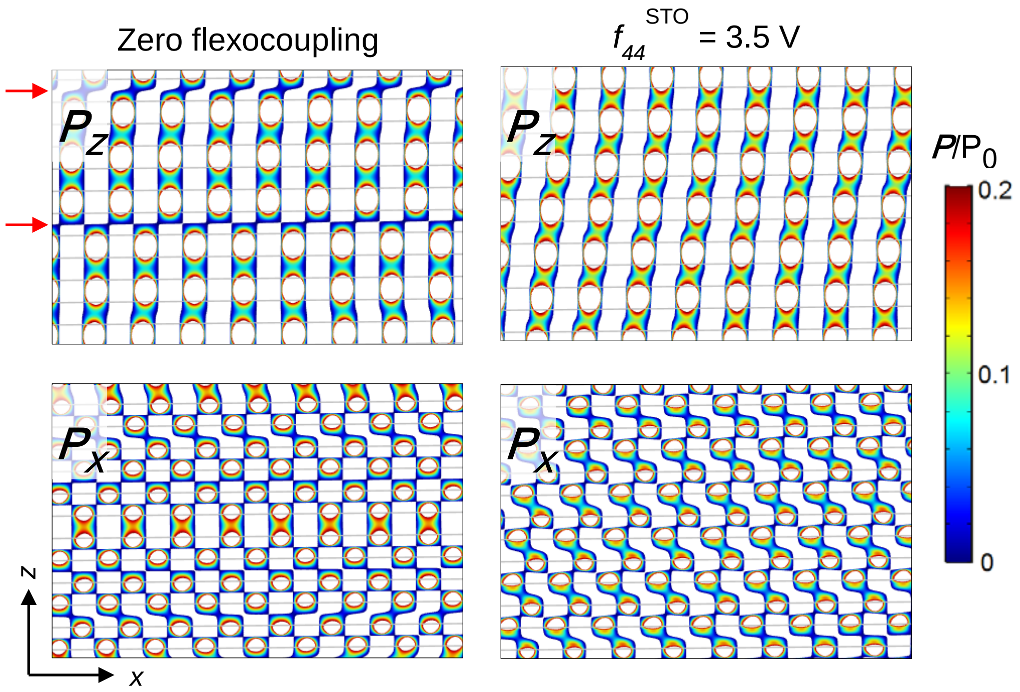

Supplementary Figure 4 | Simulated vortex arrangement under zero flexocoupling and f_{44}^{STO} = 3.5 V conditions. The data range is chosen to highlight STO polarization only in the positive side (P_0 = 0.7 C m^{-2}). Red arrows denote anti-phase vortex boundaries.

Supplementary Table 1 The energy coefficients of $PbTiO_3/SrTiO_3$ at 300 K and related model parameters

Landau Coeff.		Elastic Coeff.		Flexoelectric Coeff.		Electrostriction Coeff.	
α_1^{PTO}	-1.706×10^{8} $C^{-2}m^{2}N$	C_{11}^{PTO}	230 GPa	f_{11}	-6–6 V	q_{11}^{PTO}	1.142×10^{10} $C^{-4}m^{2}N$
α_{11}^{PTO}	-7.3×10^{7} $C^{-4}m^{6}N$	C_{12}^{PTO}	100 GPa	f_{12}	-6–6 V	q_{12}^{PTO}	4.90×10^{8} $C^{-4}m^{2}N$
α_{12}^{PTO}	7.5×10^{8} $C^{-4}m^{6}N$	C_{44}^{PTO}	70 GPa	f_{44}	-4–4 V	q_{44}^{PTO}	3.718×10^{9} $C^{-4}m^{2}N$
α_{111}^{PTO}	2.6×10^{8} $C^{-6}m^{10}N$	C_{11}^{STO}	330 GPa	Gradient-energy Coeff.*		q_{11}^{STO}	1.26×10^{10} $C^{-4}m^{2}N$
α_{112}^{PTO}	6.1×10^{8} $C^{-6}m^{10}N$	C_{12}^{STO}	100 GPa	G_{11}	1.038×10^{-10} $C^{-2}m^{4}N$	q_{12}^{STO}	-1.3×10^{10} $C^{-4}m^{2}N$
α_{123}^{PTO}	-3.7×10^{9} $C^{-6}m^{10}N$	C_{44}^{STO}	125 GPa	G_{44}	5.19×10^{-11} $C^{-2}m^{4}N$	q_{44}^{STO}	1.196×10^{10} $C^{-4}m^{2}N$
α_1^{STO}	2.017×10^{8} $C^{-2}m^{2}N$	Dielectric Permittivity		G_{12}	0		
α_{11}^{STO}	1.7×10^{9} $C^{-4}m^{6}N$	K_b^{PTO}	$20K_{0,\ Vacuum}$				
α_{12}^{STO}	4.45×10^{9} $C^{-4}m^{6}N$	K_b^{STO}	$20K_{0,\ Vacuum}$				

*The same gradient energy coefficients were used for both PTO and STO layers.
