# Supplementary material for: Quantification of flexoelectricity in PbTiO3/SrTiO3 superlattice polar vortices using machine learning and phase-field modeling
Source: Nat Commun. 2017 Nov 13;8:1468. doi: 10.1038/s41467-017-01733-8 (PMC5684141; doi:10.1038/s41467-017-01733-8)
Supplement: Supplementary file 3 — Description of Additional Supplementary Files [file 41467_2017_1733_MOESM3_ESM.pdf]

Description of Additional Supplementary Files

File Name: Supplementary Movie 1
Description: Time evolution of the vortex phase from a random polarization state.
