# Supplementary figures and images for: Quantification of flexoelectricity in PbTiO3/SrTiO3 superlattice polar vortices using machine learning and phase-field modeling
Source: Nat Commun. 2017 Nov 13;8:1468. doi: 10.1038/s41467-017-01733-8 (PMC5684141; doi:10.1038/s41467-017-01733-8)

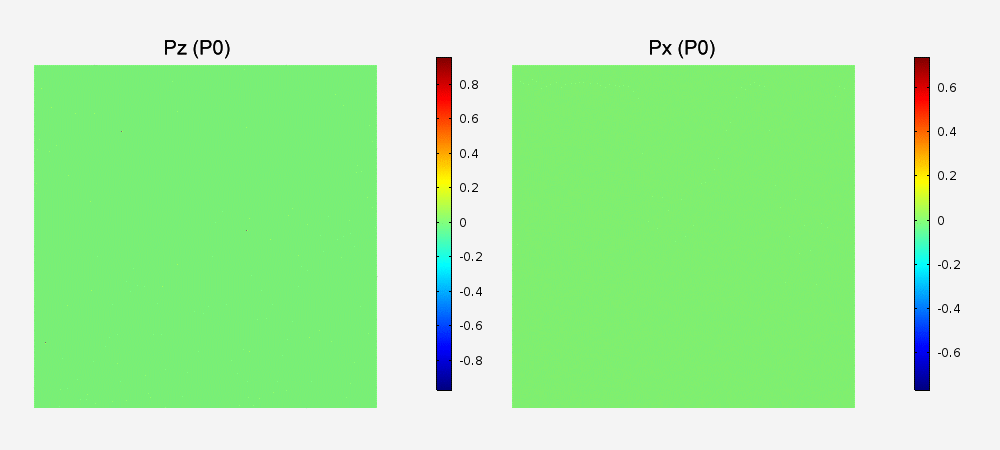

Supplement: Supplementary file 4 — Supplementary Movie 1 [file 41467_2017_1733_MOESM4_ESM.gif]
